# Large scale multi-class pest image classification using structurally adapted DenseNet architecture

**DOI:** 10.1038/s41598-026-49685-8

**Published:** 2026-04-29

**Authors:** Neetu Agrawal, Mehul Mahrishi, Mukesh Kumar Gupta, Manoj Kumar Bohra

**Affiliations:** 1 Swami Keshvanand Institute of Technology, Management and Gramothan, Jaipur, Rajasthan India; 2 National Informatics Center, Delhi, India; 3 Manipal University Jaipur, Jaipur, India

**Keywords:** Computational biology and bioinformatics, Ecology, Ecology, Environmental sciences, Mathematics and computing

## Abstract

The United Nations’ Sustainable Development Goals, SDG 12: Responsible Consumption and Production, and SDG 13: Climate Action highlight the importance of environmental conservation and reducing pesticide use. Early and accurate pest identification is essential for implementing targeted pest control measures, which helps reduce unnecessary and incorrect pesticide use. While effective pest recognition and classification are crucial for ecological research and biodiversity conservation, traditional methods remain labor-intensive, time-consuming, and dependent on experts. Several deep learning techniques have been introduced in recent years, leading to more efficient and accurate identification and classification of crop pests. This research presents a structurally adapted DenseNet model for multi-class pest image classification based on dense connections. The model is fine-tuned through hyperparameters involving dense blocks and transition layers to perform consistently across three different datasets, including the IP102 dataset, which contains over 75,000 images of 102 pest species. The study also addresses dataset imbalance to prevent biased outcomes by deep learning models. The proposed structurally adapted model for fine-grained classification achieves 82.69% accuracy and 81.45% F1 score on the IP102 dataset, complementing existing advanced methods.

## Introduction

Research by the Food and Agriculture Organization of the United Nations reveals that nearly 40% of global crops suffer damage due to pest infestations^1^. While natural and chemical pest control methods are utilized, many unaware farmers favor chemical pesticides for convenience^2^. Rather than indiscriminately applying harmful pesticides over entire fields, selectively targeting affected areas proves to be more beneficial for both environmental health and human welfare, paving the way for sustainable farming practices. To make this possible, it is essential to accurately identify pest species and implement effective pest management strategies appropriate for each specific situation^3,4^. This approach can promote a healthier agricultural ecosystem and contribute to a sustainable future, thereby aligning with the goals set forth by the United Nations.

Before automation, identifying pest species relied on manual observation, which is labour-intensive for three key reasons: it takes considerable time^5^, it demands expertise in insect taxonomy that not all farmers possess^6^, and it lacks precision because many intra-family species share similar traits^7^. Agricultural automation started with machine learning algorithms to detect pests, identify species, recognize diseases, and carry out various related tasks through image processing. However, these algorithms rely on a manual feature extraction process, which can be inefficient. Furthermore, as datasets grow in size and complexity, machine learning’s performance tends to decline, leading to subpar results^7^. Deep learning, a branch of machine learning that leverages neural networks, has dramatically influenced the automation of many tasks in different fields^3^. Convolutional neural networks have emerged as the most effective and favored model for image classification tasks^8,9^. Numerous deep learning models based on CNN have been developed and trained on extensive image datasets such as ImageNet, which is publicly accessible. After training, model parameters are saved for reuse in similar tasks with smaller datasets^10^. These pre-trained deep learning models have significantly benefited researchers, serving as a valuable resource for further advancements. In this work, we chose to work with the DenseNet model, a pre-trained CNN model with dense connections among the network layers.

Deep learning models necessitate a substantial dataset to optimize their performance^11,12^. Nevertheless, collecting pest image data presents multiple challenges. Natural barriers, inconsistent lighting, the small scale of pests, and the indistinguishable pest characteristics complicate image acquisition. Additionally, the varying prevalence of different pest species in nature is a significant concern^13^. This disparity among pest species results in a dataset characterized by a long-tailed distribution. Consequently, deep learning models can produce biased results for pest classes with more samples^14,15^. Our study used statistical methods and data augmentation to address dataset imbalance, improving results for the less-represented classes. The contributions of this research are delineated as follows:

- The pre-processing of the pest dataset to manage class imbalance to attain unbiased results through the deep learning model.
- Introduction of a structurally adapted model for fine-grained classification designed to achieve superior results for the multi-class classification task on a challenging pest dataset comprising 102 classes of pests.
- The model demonstrate robustness and generalizability when applied to multiple pest datasets, resulting in improved classification results.
- A comparative analysis of the proposed methodology against existing advanced and competitive approaches.

## Related work

Deep learning and its variants have demonstrated promising outcomes in various image classification tasks. Several researchers have explored the application of ResNet, DenseNet, and other related multi-class architectures for image classification. The relevant literature is classified into two subsections.

### Deep learning models

In^16^, the authors implemented a customized convolutional neural network to classify pest images for 3 different datasets. An accuracy of more than 95% was achieved on all datasets.^17^ adopted a transfer learning approach using VGG, ResNet, and MobileNet models with the added feature of an attention mechanism on the models to classify 14 species of citrus pests. For augmenting the dataset, an attentive recurrent GAN (Generative Adversarial Network) was used, which not only increases the image count but also does image upscaling. The average accuracy across the three models was 93.65%. In the same direction,^18^ also used a transfer learning approach, implementing various models for pest image classification with a highest accuracy of 94.64% using InceptionV3 on a pest dataset consisting of 15 species of butterflies. The authors in^19^ focused on memory-constrained devices while choosing the model for implementation. MobileNetV2 was implemented on the IP102 pest dataset, and the Cutmix method was used for image augmentation to achieve an accuracy of 71.32%. In^20^, ResNet models with self-attention were implemented on a dataset with 9 pest classes. Three ResNet models, ResNet50, ResNet101, and ResNet152, were implemented to achieve accuracies of 99.80%, 88.48%, and 96.68%, respectively. The authors in^21^ used the EfficientNet V2 model and coordinated attention mechanism for pest image classification, where EfficientNet V2 worked as the central pillar to learn about pest features and the attention mechanism to get the pest’s position in the image. The proposed approach achieved an accuracy of 98.4% on a self-constructed dataset consisting of 30 classes and 73.7% on the IP102 dataset. Many authors adopted the DenseNet model in their implementation for image classification and proved it better than many other CNN-based models.^22^ used a transfer learning approach on various CNN-based models like DenseNet201, InceptionV3, MobileNetV2, VGG19, and ResNet50. The DenseNet model achieved an accuracy of 99%, surpassing other models across 17 pest classes of jute crop. In^23^, the authors worked to identify cotton aphid pests using an enhanced DenseNet-BC-100 model. In the proposed model, each dense block was enriched with a coordinated attention mechanism to embed the location information into the channel attention mechanism. Also, the number of network parameters was reduced by decreasing 16 convolution substructures to 6 for each dense block. The resultant network achieved an accuracy of 97.3%. Authors in^24^ implemented an improved DenseNet model for classifying 10 rice pest species and achieved an accuracy of 98.28%. A Fully Convolutional Network (FCN) was used for segmentation, and DenseNet, which has an attention mechanism, was used as a feature extractor. In^25^, the authors worked on pest classification using CornerNet model. DenseNet-100 was used as the base network for feature extraction. The proposed model was implemented on the IP102 pest dataset with 102 classes to achieve an accuracy of 68.74%. A summary of the proposed approaches for image classification is shown in Table 1.

Vision transformers (ViT), introduced by^26^ in 2020, are based on the self-attention mechanism and can outperform CNNs. However, standard ViT models require large amounts of data and are computationally costly due to the self-attention mechanism. Unlike CNNs, ViT lacks spatial intelligence^27,28^ proposed the Hybrid Pooled Multihead Attention (HPMA), a modified transformer that uses Max and Min Pooling to analyse local regions, inspired by CNNs. Their streamlined model is suitable for smaller datasets (like IP102, comprising 75,000 images) and offers high accuracy despite its size. Our future endeavours include implementing ViT models to improve image classification tasks .

**Table 1:** Summary of Proposed Approaches by the aforementioned studies.

Source	Dataset	Methodology	Accuracy
^3^	5000 images of soybean pests, including 7 species	Fine-tuning & Transfer Learning Strategies on Inception-v3, Resnet-50, VGG-16, VGG-19 and Xception models	93.82%
^7^	D0 (Xie) dataset with 4500 images including 40 classes	Linear and non-linear Ensembling on 3 models.	96.18%
^10^	10 species of butterfly	Transfer Learning on VGG16, VGG19, ResNet50. VGG16 achieved highest accuracy	79.5%
^29^	IP102 & local collected pest dataset	Faster-PestNet: MobileNet as base network and improved Faster-RCNN as recognizer	82.43%
^30^	62,060 images from IP102	Model MADN combines 3 improved densnet models via ensembling	75.28%
^23^	cotton aphid pest	CA_DenseNet_BC-40 model with Coordinated attention mechanism	97.3%
^31^	D0(Xie) 40 classes, Deng 10 classes, IP102	3 high accuracy models selected for ensembling using weighted voting	Xie-98.81%, IP102-67.13%
^32^	Xie24 24 classes, D0(Xie) 40 classes, IP102	Combined fine-tuned EfficientNet for feature extraction and non-linear classifier Power mean SVM in place of linear softmax function	Xie24-99%, D0-99%, IP102-72.31%

### Related works on dataset balancing and augmentation techniques

Pests exist in an imbalanced state, with few species in abundance in a region and others difficult to find. The natural distribution of pest species is also reflected in the pest dataset yielding a long-tailed distribution^13^. The classification models which are designed to work with balanced datasets^15^, give biased results for classes containing more samples^14,33^. The classes with a greater number of samples are considered the majority classes, while those with fewer samples are classified as minority classes.

Many researchers attempted to manage dataset imbalance either at the data level or at the classification model level. Authors in^34^ used image augmentation techniques of flipping, rotation, scaling, and brightness adjustment to manage data imbalance. In^14^, the Attentive Recurrent Generative Adversarial Network (ARGAN), a data augmentation technique, was used to balance the dataset. The IP102 pest dataset was used in this work, a highly imbalanced dataset reflecting the real-life scenario of pests in nature. According to the authors, augmentation also adds to the variability of the image data, resulting in the robustness of the classification model. Authors in^33^ also adopted rotation and flipping data augmentation techniques to reduce data imbalance. The authors added 1000 samples in installments and watched the classification results until they reached an optimal stage. Authors in^15^ used resampling approaches such as random under-sampling, random over-sampling, and SMOTE (synthetic minority oversampling technique) to manage the imbalance of the two classes (noninfected and infected oil palm trees).

Many researchers also adopt a data-balancing approach based on the classification model. Transfer learning^35^ and fine-tuned models^36^ are frequently used for managing class imbalance. The learning approach is also used for the datasets with long-tailed distribution^37^. The authors in^13^ argue that the above-mentioned model-based balancing techniques are complex in designing and training the model. So, the authors looked for decoupled learning for handling data imbalance. Decoupled learning decouples feature learning from classifier training and has been part of many research works before^38^.

Various approaches have their advantages and disadvantages. For example, SMOTE generates synthetic samples by interpolating in feature space, but they can be blurry or noisy, especially for complex images like pests. Class-weighted loss assigns higher weights to minority classes, increasing their influence but risking training instability if weights are too high. Focal Loss targets hard examples, such as minority classes, but needs hyperparameter tuning. Decoupled learning involves a complex, multi-stage pipeline, while random over-sampling duplicates images to help recognize rare pest classes.

No single approach can resolve all problems^15^; therefore, it is about evaluating different approaches and models and finding the best solution. This study employed statistical techniques and an oversampling method as a pre-processing step to address class imbalance, resulting in better outcomes. Although using a simpler approach avoids the complexity of the methods discussed, it is valuable to experiment with them to discover alternative ways to handle imbalanced datasets.

## Dataset

**Figure 1:**
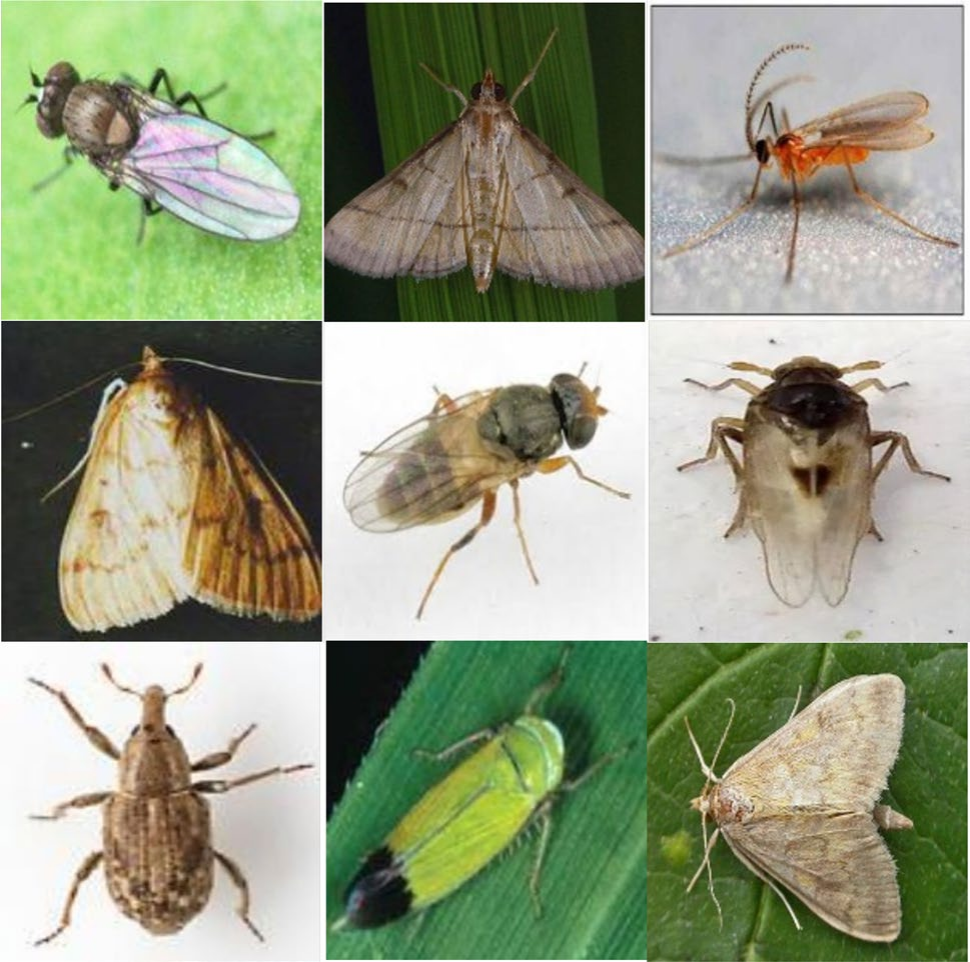
Sample images from the IP102 dataset.

We utilized the IP102 dataset in our research to validate our proposed approach. This dataset, created by^39^ in 2019, includes 75,222 images of 102 pest species that impact eight crop types, including rice, corn, wheat, beet, alfalfa, mango, citrus, and Vitis. The authors constructed a hierarchical structure to elucidate the dataset’s taxonomy. The number of species included for different crops is detailed in Table 2. Sample images from IP102 are shown in Fig. 1.

**Table 2:** Number of species for each crop in IP102 dataset.

Food crop	Number of species	Total images
Rice	14	8,417
Corn	13	14,015
Wheat	9	3,418
Beet	8	4,420
Alfalfa	13	10,390
Mango	10	9,738
Citrus	19	7,273
Vitis	16	17,551

**Figure 2:**
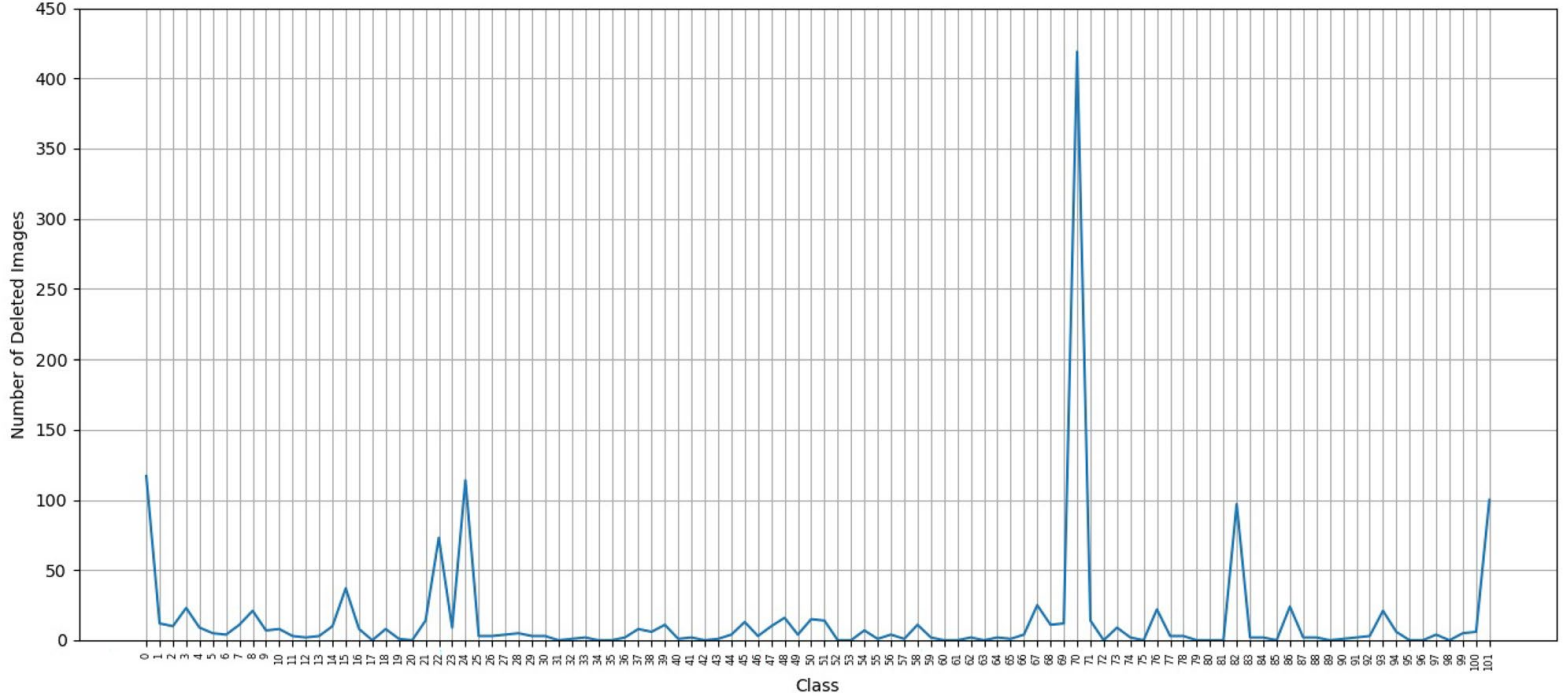
A visual overview showing the count of images deleted from each class of IP102 preprocessing.

Some species had only a few image samples, while others had a much larger number. The minimum number of samples is 71, and the maximum is 5,310. A similar pattern exists in the natural environment. Such an imbalanced dataset representing real-time scenarios is difficult to process. On average, each class has 737 samples. The number of total images was reduced to 73,747 by removing several blurred images and those lacking pest objects. The presence of unclear or blurred images within classes could potentially lead to misclassifications. Figure 2 illustrates the count of images removed from each IP102 class.

To validate our proposed structurally adapted DenseNet model, we used two more datasets: one is the Xie dataset^40^, and the other is a 9-class dataset from Kaggle. The Xie dataset (D0) consists of 4,500 images and 40 species of agricultural pests affecting mainly 4 crops (corn, soybean, wheat, and canola). The 9-class dataset consists of 9 pest species, including aphids, armyworms, beetles, bollworms, grasshoppers, mites, mosquitoes, sawflies, and stemborers, with 3,150 images.

### Dataset balancing and augmentation

Imbalanced class distributions cause the deep learning model to produce biased classification results, favouring the majority classes. We applied a statistical method to detect minority classes and balance the dataset. IP102 is an inherently imbalanced dataset, so it was chosen to verify the proposed dataset balancing approach. Statistical techniques are vital in data analytics and machine learning. Specifically, the median statistic was chosen to isolate minority classes. The dataset was divided into training, validation, and test sets. Balancing and augmentation were performed only on the training set to prevent data leakage. Test set consists of original unaugmented images. The classes in the training set were sorted in increasing order of the image count. The list of image counts was then used to calculate the median, as shown in equation (1). Here, n indicates the total number of classes. Because the list contains an even number of items, the median is found by taking the average of the two central values.

1 $$\begin{aligned} \begin{aligned} \text {Median for Even number of items}=&\quad \frac{\left( \frac{n}{2}\right) ^\text {th} value + \left( \frac{n}{2}+1\right) ^\text {th} value}{2} \quad \\ \text {Median for Odd number of items}=&\quad \left( \frac{n+1}{2}\right) ^\text {th} value \quad \end{aligned} \end{aligned}$$

Classes with image counts below the median were considered minority classes. These minority classes were oversampled using geometric transformation augmentation techniques, including rotation, vertical flipping, translation, and adjustments to brightness and contrast. Geometric transformations help the model train on pest images regardless of their orientation or spatial changes, simulating real-life pest scenarios. For CNN models like DenseNet, a geometrically transformed image is considered a different input because the spatial relationships between pixels change relative to the coordinate system.

The augmentation of minority classes was carried out in multiple steps. The process was repeated until the class imbalance was sufficiently reduced to improve classification results. To assess the dataset’s optimality, the augmented data was tested at each intermediate stage across several deep learning models, such as InceptionV3, MobileNetV2, and DenseNet121. The adopted approach resulted in a balanced version of IP102, called BIP102. The balanced dataset contains approximately 110,000 images. In summary, the original IP102 dataset contains 75,222 images with a long-tailed distribution. There is substantial variance in the number of images across classes, with a minimum of 71 and a maximum of 5,310. The BIP102 dataset includes 110,000 images with less imbalance among the classes. Most classes have image counts in the range of 700-900, thus preventing biased results.

**Table 3:** Number of images in a few classes before and after balancing them.

Class/specie	Original IP102 dataset	BIP102 dataset
Rice Leaf Roller	730	730
Paddy Stem Maggot	148	730
Mole Cricket	1430	1430
Wireworm	550	1100
Peach Borer	496	992
English Grain Aphid	315	1260
Beet Army Worm	1058	1058
Beet Spot Flies	225	1116
Locustoidea	1164	1164
Alfalfa Seed Chalcid	153	750

**Figure 3:**
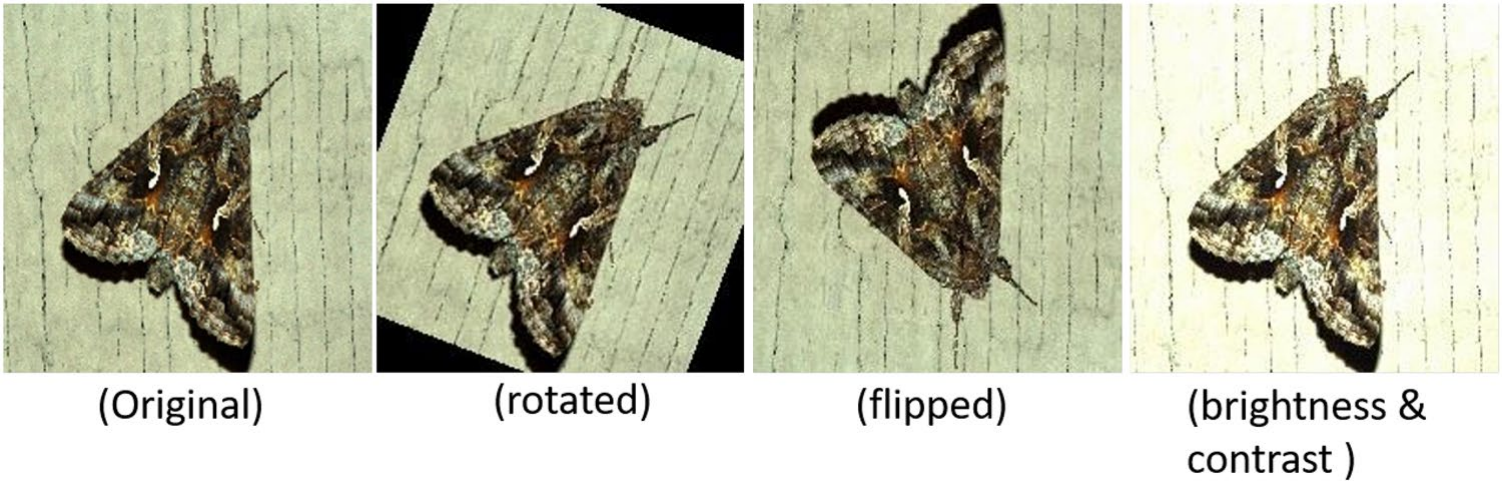
Sample images after applying Geometric transformations for Augmentation.

Table 3 displays the image count for selected classes in original imbalanced dataset and the processed balanced dataset. Some of the classes which were not part of the minority group were not augmented. Sample augmented images of one of the minority classes are displayed in the Fig. 3.

## Proposed methodology

There are numerous pre-trained CNN models available for image classification tasks. We identified frequently used pest classification models from related works by various authors, such as MobileNetV2, InceptionV3, DenseNet121, DenseNet201, ResNet50, and VGG19. The different attributes of the models include size, parameters, and depth. Models with numerous parameters are resource-intensive, resulting in high training times. However, lightweight models like MobileNetV2 are designed to operate on memory-constrained devices. Different CNN models may yield varying results based on the nature of the dataset. For the current study, the DenseNet model was selected as the base model, striking a balance between the number of model parameters, size, and training time.

**Figure 4:**
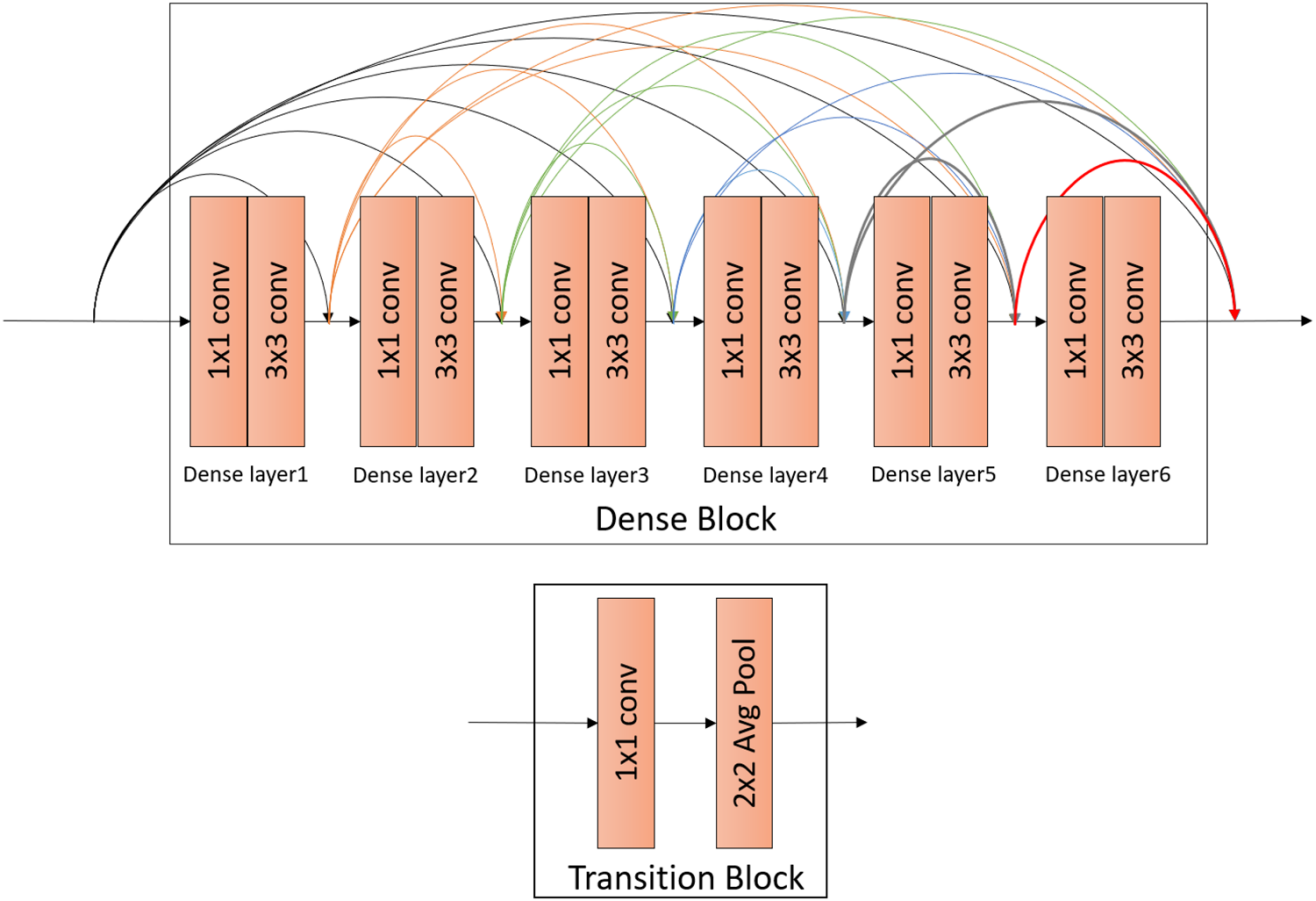
DenseNet architecture with one dense block consisting of 6 dense layers and a transition block.

The DenseNet architecture includes primarily dense blocks, transition blocks, and a classification head. Each dense block comprises multiple layers; each layer directly receives feature maps from all preceding layers. This dense connection between the network layers allows for better feature reuse in fewer parameters^30^ and better feature representation. This characteristic helps reduce overfitting, shorten training time, and limit hardware resource requirements. Better feature representation supports working with limited training time. However, as there are dense connections, it can be computationally expensive. Figure 4 shows one dense block of a DenseNet model. Between each dense block, there is a transition block that performs downsampling and reduces feature maps. A transition block consists of a 1x1 convolutional layer and an average 2x2 pooling layer. After the last dense block, there is a global average 7x7 pooling layer. The resultant feature maps from this pooling layer are flattened and connected to a fully connected layer for classification.

### Proposed deep learning model

**Figure 5:**
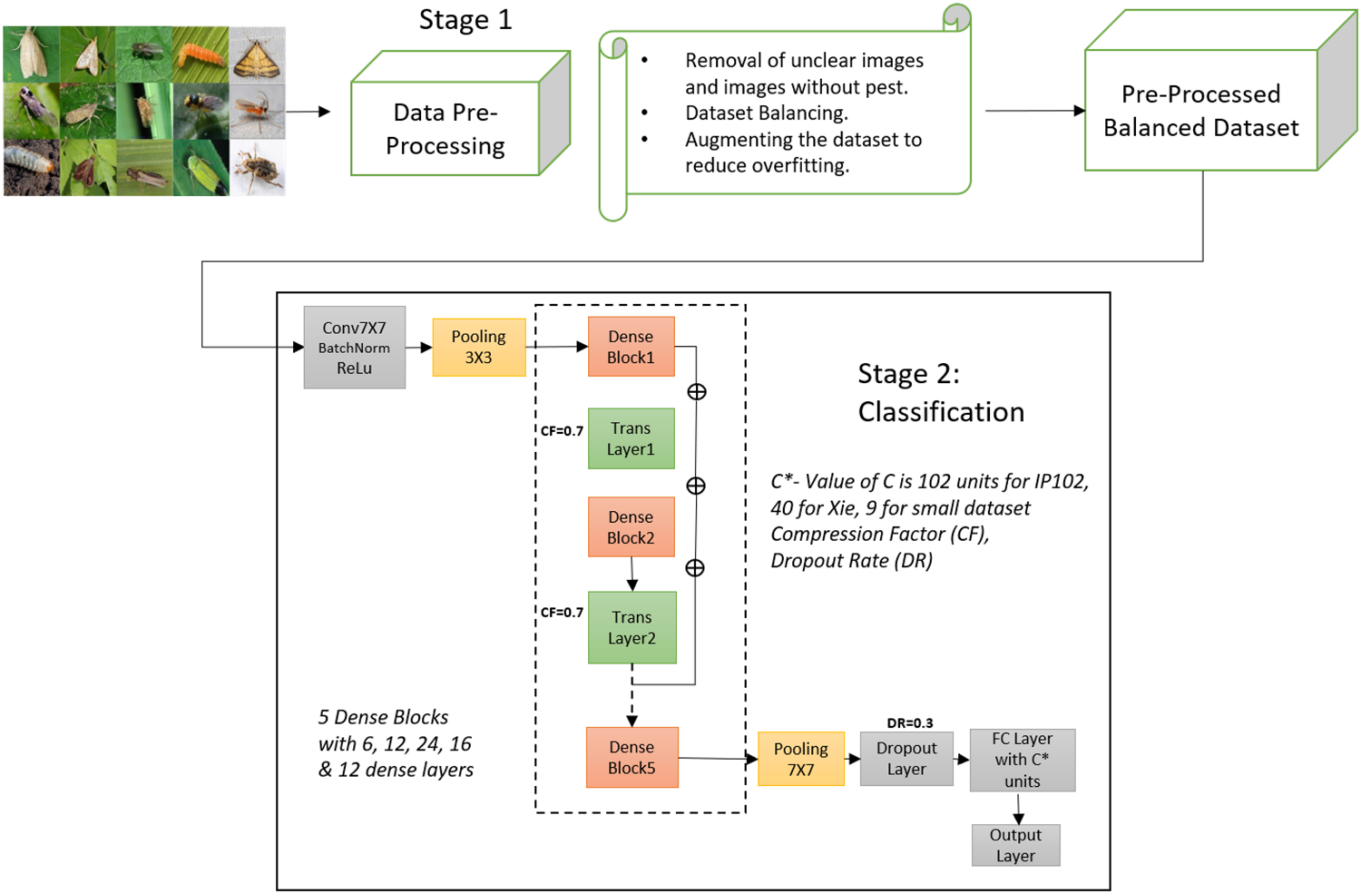
Proposed methodology including dataset balancing and structurally adapted DenseNet model.

The current study employed DenseNet as the base model and introduced a structurally adapted DenseNet model for fine-grained image classification. The base model was frozen, thus providing a stable and reliable starting point, with only the new blocks added and trained. This approach directs learning towards the structural differences important for agricultural pests or any fine-grained image classification. The framework of our proposed methodology including dataset balancing is as demonstrated in Fig. 5.

We modified the model by adding a dense block and a corresponding transition block in the design, which increased the depth of the model. An increase in depth results in a more robust model, but at the cost of adding to model complexity and overfitting. So, we conducted experiments to determine the number of dense blocks to add. The increase in the number of convolution layers in a dense block increases feature learning capacity while simultaneously increasing the model’s computing complexity, which may eventually lead to no improvements in accuracy. After some experimentation, a balance was required, so we included 12 layers in the added Dense block.

Corresponding to the new Dense Block, a Transition Block has also been added. The role of transition blocks in the model is crucial. They decimate the spatial dimension and compress the feature maps. An attribute in the transition layer, the compression factor, regulates the number of feature maps. It determines the number of retained feature maps and others left behind after each dense block. The value of the compression factor is between 0 and 1, the suggested value being 0.5. A compression factor with a value of 1 means all feature maps are retained while maintaining more information about the features of the network. But higher compression factor could also lead to a more complex model with increased computational costs. The optimal value of the compression factor in a given scenario also relies on the dataset and the performance desired from the model. Let us denote the compression factor with theta ($\theta$). Suppose the number of feature maps as input to the transition block is $X_{in}$ and the number of output feature maps from the transition layer is $X_{trans}$. The value of $X_{trans}$ can be calculated as,

2 $$\begin{aligned} X_{trans} = \theta * X_{in} \end{aligned}$$

The output from the transition block becomes the input to the next dense block. Suppose the subsequent dense block consists of L layers and a growth rate of K. Each layer will add K feature maps to the dense block, corresponding to the growth rate. Therefore, the growth rate and the number of layers influence the number of output feature maps and parameters. For this implementation, the default growth rate of 32 is used. The output feature maps from this dense block can be calculated as

3 $$\begin{aligned} X_{out} = X_{trans} + K *L \end{aligned}$$

**Figure 6:**
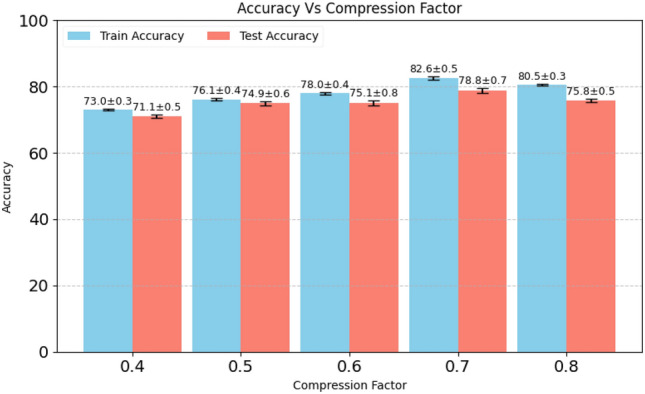
Train and test accuracies for various compression factor values.

It is clear that the compression factor directly influences the number of feature maps passed as input to the subsequent dense block. Identifying the appropriate compression factor requires experimentation with various values, assessing accuracy results to determine the optimal setting. We conducted a systematic grid search over a range of compression factor values [0.4, 0.8] across five separate experiments. A lower compression factor (0.4) resulted in a slight decrease in maximum accuracy. Beyond a value of 0.7, overfitting increased along with higher memory consumption. Standard deviations were determined through five independent runs per configuration. The small margin of error across these runs indicates that the performance improvements are statistically robust. Figure 6 presents the chart for accuracy outcomes associated with different compression factor values. We opted for a compression factor value of 0.7 with improved results for train and test accuracy maintaining a balance between model complexity and model performance.

Through the calibration of the fifth dense block and adjusting the compression factor, we have transformed the standard DenseNet model into a specialist. We constructed an architectural bridge that transforms general ImageNet features into specialised fine-grained image data, by enabling the model to develop high-level abstraction layers. In domains where classes are 99% identical (such as two types of pests), standard models tend to be too shallow. By systematically incorporating abstraction layers (the fifth dense block) and enhancing feature retention via an adjusted compression factor, this methodology can be applied to any visual task where the preservation of minute morphological details is essential for classification accuracy. The structurally adapted DenseNet as proposed, provides a framework for other fine-grained classification tasks characterized by long-tailed distributions.

Considering the current research area, pest species often show high similarity within the same class across growth stages and low variance between different species. Standard DenseNet models like densenet121, densenet169 and densenet201 (comprise 4 dense blocks with different number of dense layers in each) tend to experience feature dilution in deeper layers. Incorporating a Dense Block promotes deeper feature concatenation, keeping low-level morphological details accessible to the final classifier. Consequently, our model, built on the standard DenseNet architecture, is engineered to overcome the challenges of classifying insect images.

A dropout layer is added before the fully connected layer (FC layer). The dropout rate was selected following experimentation with various values. We conducted a systematic grid search over a range of dropout rates [0.2, 0.5] across four separate experiments. We observed the difference between training and validation accuracy for each run. For lower dropout rates, training accuracy exceeded validation accuracy, indicating a high generalisation gap and early signs of overfitting. At higher dropout rates, training showed slower convergence and achieved lower maximum validation accuracy. This suggests that the model becomes too limited to learn the actual features. At a dropout rate of 0.3, the gap between training and validation accuracy was acceptable (around 4%). The same pattern was observed in training and validation loss values. Consequently, we selected a dropout rate of 0.3.

**Figure 7:**
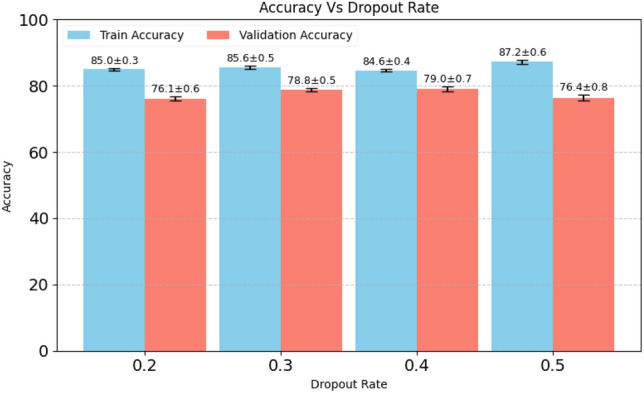
Train and validation accuracies for various dropout rate values.

Standard deviations were determined through five independent runs per configuration. The small margin of error across these runs indicates that the performance improvements are statistically robust. This is summarized in the chart in Fig. 7. The enhanced model comprises approximately 9M parameters, resulting in a more efficient feature extraction process and substantially increased model performance. A summary of the proposed DenseNet model layers is shown in Table 4. Transition Layer 4 and Dense Block 5, with 12 layers, are the added functionalities to the original DenseNet121 model. The compression factor value is maintained at 0.7 for the transition layer. A dropout layer is added before the classification layer with a dropout rate of 0.3 to mitigate overfitting. The table 5 presents the ablation study of the proposed model on the BIP102 dataset to demonstrate the effectiveness of each component.

**Table 4:** Proposed DenseNet layer details with 5 Dense Blocks, 4 Transition Layers, and a Dropout Layer.

Layer	Input dimension	Operation	Description
initial convolution	224$\times$224	7$\times$7 conv, stride 2	initial feature extractor
max pooling	112$\times$112	3$\times$3 max pool, stride 2	spatial downsampling
Dense Block 1	56$\times$56	6 dense layers	concatenated feature maps
Transition Layer 1	56$\times$56	1$\times$1 conv and Avg Pool	Compression & Downsampling
Dense Block 2	28$\times$28	12 dense layers	–
Transition Layer 2	28$\times$28	1$\times$1 conv and Avg Pool	–
Dense Block 3	14$\times$14	24 dense layers	–
Transition Layer 3	14$\times$14	1$\times$1 conv and Avg Pool	–
Dense Block 4	7$\times$7	16 dense layers	–
Transition Layer 4	7$\times$7	1$\times$1 conv and Avg Pool	Compression & Downsampling
Dense Block 5	4$\times$4	12 dense layers	added to optimized architecture
Global Avg Pool	4$\times$4		Feature Map reduces to 1$\times$1
Dropout	1$\times$1		Reduces Overfitting
Classification	1$\times$1	softmax Layer	Output Prediction

**Table 5:** Ablation study of the proposed model.

Method	Training loss	Train accuracy (%)	Validation loss	Validation accuracy (%)
DenseNet121+ One DB	0.4807	0.8686	0.8095	0.8028
DenseNet121+ One DB+CF	0.4790	0.8687	0.7822	0.8105
DenseNet121+ One DB+CF+DR	0.5063	0.8682	0.7674	0.8269

DB: Dense Block, CF: Compression Factor, DR: Dropout Layer.

## Experiments and results

### ADAM optimizer

The ADAM optimizer was utilized in this study because it is widely favored in different deep-learning classification tasks. It uses an adaptive learning rate, adopting different learning rates for sparse and dense feature parameters. ADAM is the combination of AdaDelta and SGD with momentum optimizers. It takes advantage of adaptive learning rate and momentum values. As a result, ADAM is a better choice when the data is complex and the model has many parameters. ADAM updates model parameters with the help of past and recent gradients. Two vectors, m and v, are defined to manage the moving average of gradients and the moving average of squared gradients. The vectors m and v are named as first-moment and second-moment vectors, respectively. The first step is to compute the gradient for each iteration as given by equation (4).

4 $$\begin{aligned} g_{t} = \nabla _{\theta }f_{t}(\theta _{t}-1) \end{aligned}$$

Here, $g_{t}$ is the gradient for iteration t.

$\nabla _{\theta }$ is gradient for model parameters $\theta$,

$f_{t}$ is the objective function that was optimized (maximize or minimize for a specific problem) for the previous iteration $\theta _{t-1}$

The next step is to compute the values of the first moment and the second moment m and v. The first moment is computed using Eq. (5), and the second moment is computed using Eq. (6).

5 $$\begin{aligned} m_{t} = \beta _{1}\cdot m_{t-1}+(1-\beta _{1})\cdot g_{t} \end{aligned}$$

6 $$\begin{aligned} v_{t} = \beta _{2}\cdot v_{t-1}+(1-\beta _{2})\cdot g_{t}^2 \end{aligned}$$

Here $m_(t-1)$ is the first-moment vector at time t-1, $v_(t-1)$ the second-moment vector at time t-1, $\beta _1$, $\beta _2$ are the exponential decay rate. At iteration t=0, first-moment and second-moment vectors are initialized to 0. So, they tend to be biased towards 0 for the initial few iterations, which mandates correcting them before updating model parameters. Decay rates, $\beta _1$, $\beta _2$, are used for correction. Mathematical equation for the same are (7) and (8). $\hat{m}_{t}$ is the corrected first-moment vector, and $\hat{v}_t$ is the corrected second-moment vector.

7 $$\begin{aligned} \hat{m}_{t} = \frac{m_t}{1-\beta _1^t} \end{aligned}$$

8 $$\begin{aligned} \hat{v}_{t} = \frac{v_t}{1-\beta _2^t} \end{aligned}$$

Finally, the updated model parameters at iteration t+1 can be computed using Eq. (9). Here $\alpha$ is the learning rate, and $\epsilon$ is a small value to avoid division by zero.

9 $$\begin{aligned} \theta _{t+1}= \theta _{t}-\frac{\alpha \cdot \hat{m_t}}{\sqrt{ \hat{v_t}}+\epsilon } \end{aligned}$$

Therefore, parameters at each iteration depend on both first-moment and second-moment values. It takes care of both the momentum of gradients and the variation in gradients, leading to better optimization.

### Experimental setup

The model training was conducted on a Mac M1 Pro having a 10-core CPU and a 16-core GPU, supported by 16 GB of RAM. We implemented various components of our deep learning model using Python, along with the TensorFlow framework and Keras libraries. The images were resized to 224$\times$224 before training the model. Normalization was applied using ImageDataGenerator. The proposed model utilised the ADAM optimiser to leverage adaptive learning rates during training with initial learning rate of 0.0001. It employed the Sparsecategoricalcrossentropy loss function, and to address overfitting and underfitting, an L2 kernel regularizer with a value of 0.01 was applied. Small batch size of 32 was used and the model was trained for 20 epochs. The dataset were split into training, validation, and test sets in the ratio of 6:3:1. Balancing and augmentation methods were used on the training set for IP102 dataset. Test set images were reserved as unseen data.

ImageNet pre-trained weights were used and the base layers were frozen, training only the added blocks that helped to keep a stable foundation. The original DenseNet weights are excellent at detecting basic features like edges, textures, and colors. Since only a small fraction of the 9.4 million parameters were updated, the model cannot simply memorize the training data, which reduced overfitting.

### Performance evaluation

**Table 6:** Comparison of model implementations on the various datasets used in this work.

**Model**	**Accuracy (%)**	**Precision (%)**	**Recall (%)**	**Avg.F1-Score(%)**
Proposed Model +IP102	78.76	78.0	78.76	77.0
Proposed Model +BIP102	82.69	82.58	82.28	81.45
D0(Xie)	98.97	98	97.0	97.20
9-class	99.52	99.0	98.0	98.50

The introduced methodology focuses on optimizing the hyperparameters inherent to the DenseNet framework, such as the number of dense blocks and transition layers, the number of dense layers within each dense block, the compression factor, and the dropout rate, aimed at effectively mitigating model overfitting. The proposed strategy was executed on both the original imbalanced dataset and the balanced dataset. Notably, the approach yielded improved outcomes when applied to the balanced dataset, achieving an accuracy rate of 82.69%. Additional metrics are encapsulated in Table 6. The proposed structurally adapted DenseNet model was also implemented on the D0(Xie) and 9-class datasets. The results are as shown in Table 6. Since these datasets have fewer classes and image samples, a learning rate of 0.00001 and a dropout rate of 0.4 were selected to prevent overfitting. No balancing techniques were applied to either dataset. The implementation primarily aimed to demonstrate the effectiveness of the proposed model.

**Figure 8:**
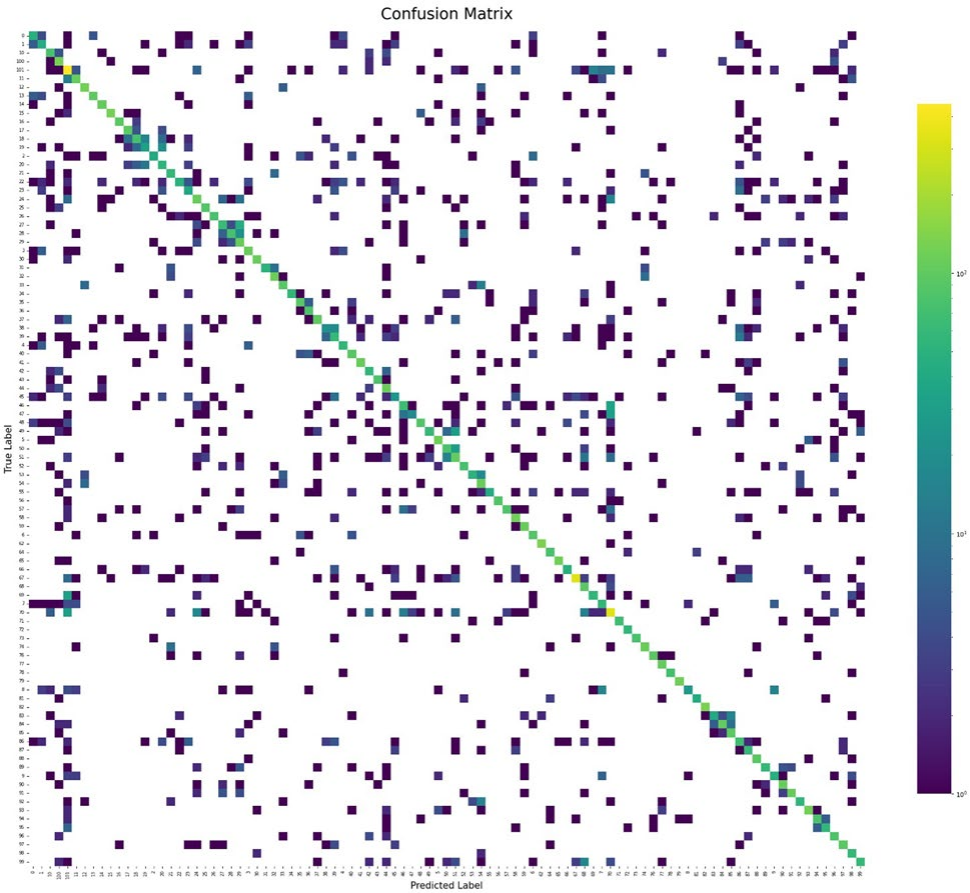
Confusion matrix of proposed model on balanced IP102 dataset.

As the IP102 dataset is not completely free of imbalance, performance metrics like the confusion matrix, performance of each class and precision-recall curves will provide better insight.The heat map of the confusion matrix in Fig. 8 assesses the validation dataset for BIP102 with the proposed method. Bright diagonals indicate high accuracy and the True Positives where model’s prediction matches the actual class. Bright line signifies that the model is learning the distinct features of most classes. The bright off-diagonals reveal errors, the misclassifications. Those classes are visually too similar for the model. Dark purple squares away from the diagonal represent random errors or edge cases, and those near the diagonal indicate misclassifications among similar classes. Overall, a bright diagonal combined with scattered purple squares indicates strong performance.

**Figure 9:**
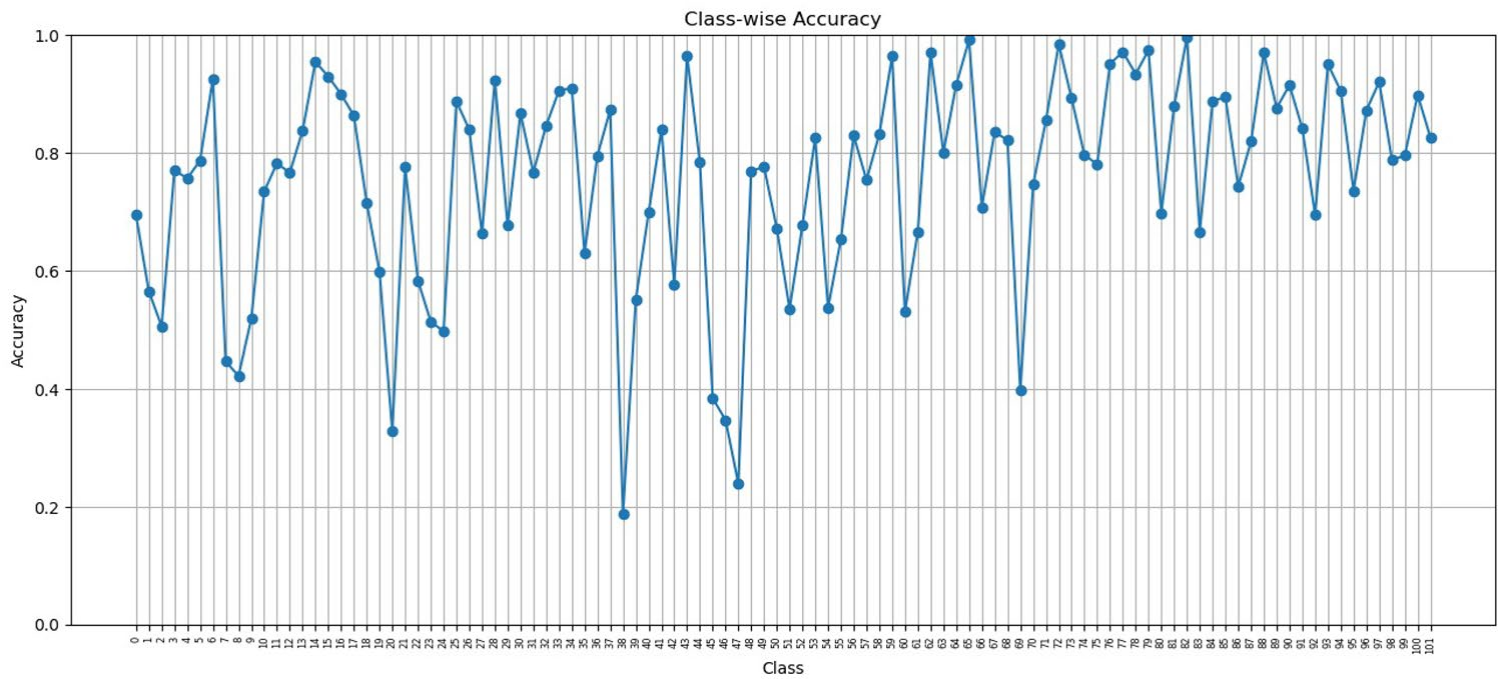
Class-wise performance of proposed model on balanced IP102 dataset.

Class-wise performance of the proposed model on BIP102 dataset is shown in Fig. 9. As can be seen, classes 20, 38, 45, 46, 47, and 69 show a significant dip in performance. The reasons are the presence of imbalance and high similarity among the classes.

**Figure 10:**
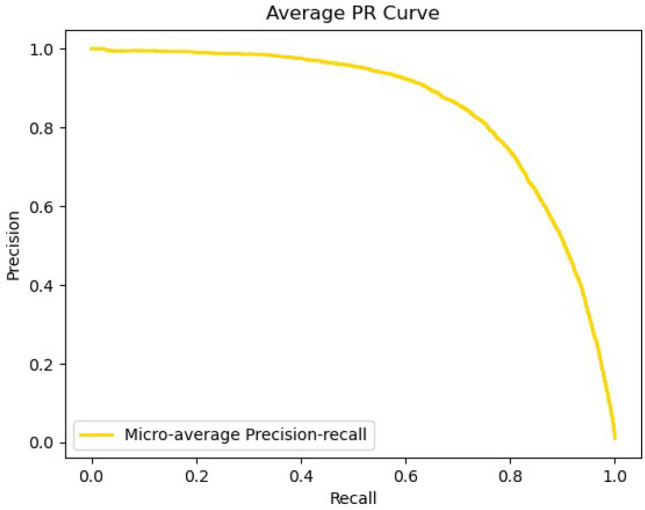
Precision-recall curve of the proposed DenseNet model on BIP102 dataset.

Figure 10 shows the precision-recall curve for the performance of the model. Examining the graph where Recall is approximately 0.80, the Precision is also around 0.75-0.80, indicating a balanced model. It is not “over-predicting” classes (high recall/low precision) nor being “too conservative” (high precision/low recall). For a significant portion of the data (up to about 0.6 Recall), precision remains very high, exceeding 0.95. This suggests that for 60% of the images, the model is almost always correct. As recall approaches 1.0, Precision declines rapidly. This indicates the model is uncertain about the final 15–20% of images in each class, which are probably blurry, dark, or difficult images that resemble other classes.

**Figure 11:**
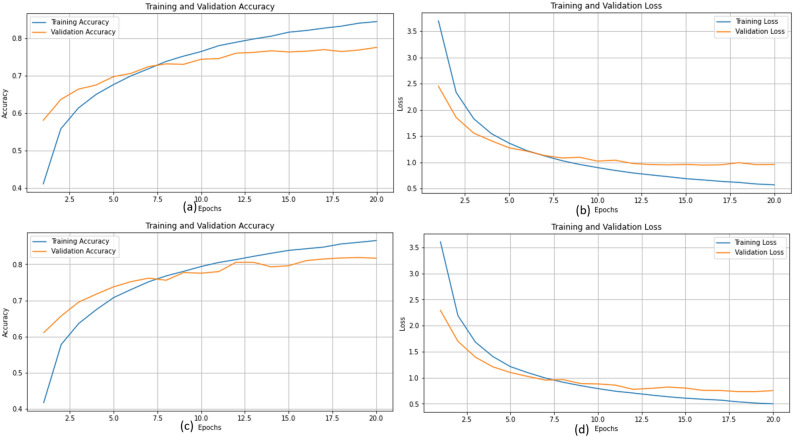
Accuracy and Loss graphs of Proposed DenseNet model on (**a**,**b**) Imbalanced IP102 dataset and (**c**,**d**) Balanced BIP102 dataset.

Furthermore, the accuracy and loss graphs corresponding to the proposed model across both datasets are illustrated in Fig. 11. As can be seen from the graphs, the gap between training and validation accuracy has decreased for BIP102 dataset. The balanced classes and data augmentation has reduced overfitting. The similar situation is visible for the graphs between training and validation loss.

Table 7 compares the model with existing approaches regarding accuracy. The DenseNet model used in this work is efficient in terms of parameters but requires more memory due to its architecture. Compared to lightweight models like MobileNetV2 and EfficientNetB0, DenseNet121 has more parameters (8.2M), a larger size (31.23MB), and is slower (higher inference time) because it uses concatenation. It performs approximately 2.8G FLOPS to process a single image. Nevertheless, for pest classification, where identifying fine details of insects is crucial,the DenseNet’s feature reuse makes it better at differentiating similar inter-class features.

The inference time of the proposed model, which is the average duration required to process a single image with a batch size of 32, is 3.4 milliseconds. With a storage footprint of only 36 MB and a computational requirement of approximately 3.1 gigaflops, the model remains significantly below the thresholds typically necessary for real-time mobile and UAV (Unmanned Aerial Vehicle) applications. In conclusion, our model is appropriate for deployment on contemporary edge devices (with 2GB to 8GB of RAM), offering an optimal balance among processing speed, classification accuracy, and the capacity to differentiate pests with similar morphologies.

**Table 7:** Comparison of related works and the proposed approach on the IP102 dataset.

Related works	Model	Accuracy (%)
Wu et al.^39^	ResNet50 & ML models	49.5
Ren et al.^41^	FR-ResNet (Stacking of Feature-reuse residual blocks)	55.24
Nanni et al.^42^	Ensembling of ResNet50, GoogleNet, DenseNet201, MobileNetv2, ShuffleNet	61.93
Ayan et al.^31^	Ensembling of 3 pre-trained CNN models	67.13
Doan et al.^32^	EfficientNet-B4, EfficientNet-B5	72.31
Ung et al.^43^	Ensembling of ResNet50, RAN, FPN, and MMAL-Net	74.13
Lin et al.^44^	Hierarchical Complementary Network(ConvNext-B as backbone network)	75.36
Ali et al.^29^	Faster-PestNet: MobileNet as base network and improved Faster-RCNN as recognizer	82.43
Proposed approach	Structurally adapted DenseNet	**82.69**

## Conclusion and future directions

Research indicates that deep learning algorithms, particularly convolutional neural networks have attained unprecedented advancements in the realm of image processing. While initial machine learning models showed promise, challenges such as manual feature extraction and dataset complexity emerged. Challenges also arise in pest classification tasks, primarily due to the inherent limitations of collecting pest image data, like diminutive sizes of pests, background noise, and the complexities of diverse terrains. This study focuses on enhancing pest classification by developing a structurally adapted DenseNet model for fine-grained classification and evaluating its performance against existing state-of-the-art methodologies. The proposed model, benefiting from a robust framework, demonstrates improved generalizability and classification results across various pest datasets. Extensive experimentation with model parameters, including dropout rates and compression factors, indicates that the devised model is well-suited for real-world applications, offering potential for real-time pest classification in modern agricultural settings while aligning with the United Nations’ sustainable goals. Furthermore, the approach includes dataset balancing and enhancement of the benchmark dataset IP102. As a result, a significant improvement in image classification performance is observed.

In our work, we tested the proposed approach on three pest datasets. To assess its generalization ability, we plan to evaluate it on a cross-domain dataset in future efforts. Furthermore, techniques like SMOTE, Focal Loss, and Class-Weighted Loss have not been implemented yet but are planned for future development. Additionally, advanced models such as Vision Transformer have shown impressive results in existing studies and are also part of our future research.

Although the proposed architecture outperforms for large-scale multi-class pest classification, several directions remain for further investigation. Firstly, to rigorously assess model robustness and generalization, future studies can evaluate the framework on cross-domain and unseen datasets captured under diverse field conditions, including variations in illumination, occlusion, and background complexity. Secondly, techniques such as Synthetic Minority Over-sampling Technique (SMOTE), Focal Loss, and Class-Weighted Loss can be adopted to address class imbalance inherent in large pest datasets. Third, while the DenseNet backbone effectively promotes feature reuse and gradient propagation, recent advances in Vision Transformers (ViTs) and hybrid CNN–Transformer architectures have demonstrated superior capability in modeling long-range dependencies. Comparative analyses with such architectures can be conducted to explore potential performance gains. Furthermore, while the current study aims to maximize classification accuracy through structural optimization, our model serves as a foundational architecture and can be extended as a feature extraction backbone for object detection frameworks, such as YOLO and its variants. Integration with edge-enabled platforms–such as smart traps based on the Internet of Things (IoT), mobile devices, and drone-mounted imaging systems can be pursued to enable real-time, on-site detection monitoring.

## Data Availability

The dataset used during the current study is publicly available and its source has been mentioned in the manuscript
